# Hypertensive Heart Disease: Mechanisms, Diagnosis and Treatment

**DOI:** 10.31083/j.rcm2503093

**Published:** 2024-03-06

**Authors:** Xuewei Huang, Lizhi Hu, Zhuojun Long, Xinyao Wang, Junru Wu, Jingjing Cai

**Affiliations:** ^1^Department of Cardiology, The Third Xiangya Hospital, Central South University, 410013 Changsha, Hunan, China; ^2^Xiangya School of Medicine, Central South University, 410013 Changsha, Hunan, China

**Keywords:** hypertensive heart disease, mechanisms, diagnosis, treatment

## Abstract

Hypertensive heart disease (HHD) presents a substantial global health burden, spanning a spectrum from subtle cardiac functional alterations to overt heart failure. In this comprehensive review, we delved into the intricate pathophysiological mechanisms governing the onset and progression of HHD. We emphasized the significant role of neurohormonal activation, inflammation, and metabolic remodeling in HHD pathogenesis, offering insights into promising therapeutic avenues. Additionally, this review provided an overview of contemporary imaging diagnostic tools for precise HHD severity assessment. We discussed in detail the current potential treatments for HHD, including pharmacologic, lifestyle, and intervention devices. This review aimed to underscore the global importance of HHD and foster a deeper understanding of its pathophysiology, ultimately contributing to improved public health outcomes.

## 1. Introduction

Despite notable advancements in blood pressure management, hypertension remains a prevalent health condition, particularly in developing countries, with low control rates [1, 2]. Sustained elevated arterial blood pressure imposes an increased cardiac load, triggering a cascade of structural and functional changes in the heart known as hypertensive heart disease (HHD) [3]. The disease burden caused by HHD has risen steadily in recent years, and HHD was the main cause of 1.16 million deaths in 2019 worldwide [4]. A common manifestation of HHD is left ventricular hypertrophy (LVH) [5]. Findings from large-scale population studies based on echocardiograms indicate that the prevalence of LVH among individuals with hypertension exceeds 20% [6] and is significantly higher in Asians and Africans [7, 8]. However, in addition to race, the detection rate of LVH also greatly depends on factors such as age, sex, and the diagnostic methods employed [9, 10]. Patients with HHD face a significant risk of progressing to heart failure (HF) [11, 12, 13]. Thus, HHD is a spectrum, ranging from asymptomatic left ventricular diastolic dysfunction to clinical heart failure [14]. Additionally, HHD has been linked to numerous adverse cardiovascular outcomes, including myocardial infarction, stroke, and even sudden cardiac death (SCD) [10, 15, 16].

In recent decades, the comprehension of HHD has transformed. Initially, viewed as a cardiomyocyte response to increased load, it is now recognized as a complex and dynamic phenomenon encompassing structural and functional changes in the heart [17]. These changes arise from various factors, such as neurohormones, myocardial metabolic remodeling, interstitial fibrosis, immunity, inflammation and mechanosensation [18, 19]. Advancements in treatment strategies have significantly impacted the prognosis of individuals, with new protocols for blood pressure management, particularly intensive antihypertensive approaches, yielding promising results [20, 21]. A notable breakthrough in combating heart failure is the utilization of sodium-glucose cotransporter-2 (SGLT2) inhibitors, which have proven effective in reducing left ventricular mass in participants with LVH and type-2 diabetes [22]. Furthermore, novel potentially therapeutic tools based on an improved understanding of the mechanisms underlying HHD have been proposed, offering potential avenues for intervention [23, 24]. This article summarizes the mechanisms associated with HHD, delves into the dynamic progression of the disease across different stages, and reviews past and future therapeutic tools.

## 2. Mechanisms Contributing to Hypertensive Heart Disease

**Fig. 1.**
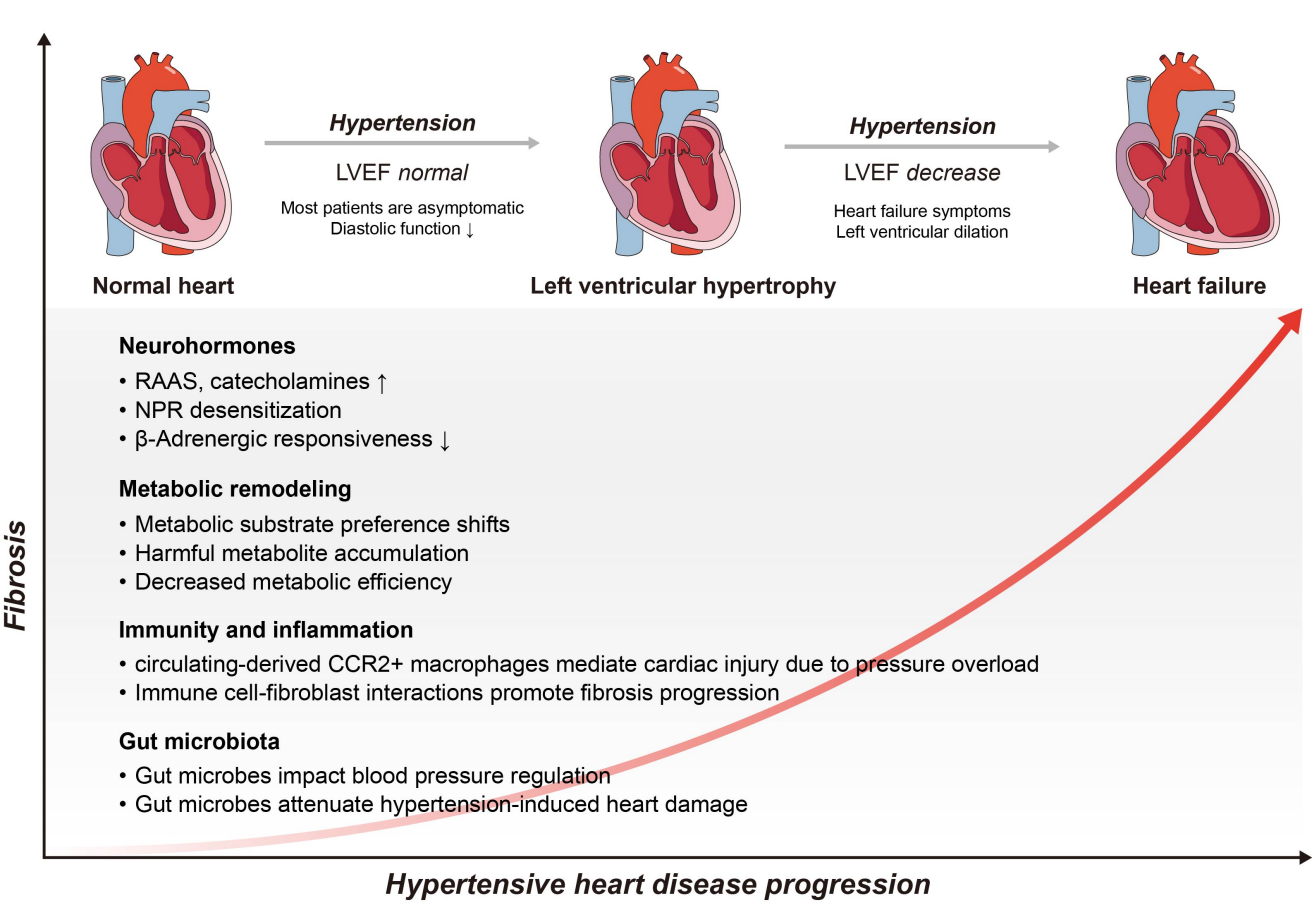
**Complex mechanisms of hypertensive heart disease (HHD)**. As HHD progresses, the level of cardiac fibrosis increases, while the heart has different macroscopic manifestations. Multiple factors, including neurohormones, metabolic remodeling, inflammation, and gut microbiota, among others, contribute to this progression. LVEF, left ventricular ejection fraction; NPR, natriuretic peptide receptors; RAAS, renin-angiotensin aldosterone system.

When hypertension leads to increased cardiac load, the ventricular wall thickens initially to reduce stress and maintain contraction efficiency, following Laplace’s law [25]. Cardiomyocytes cannot proliferate, so ventricular wall thickening occurs through a parallel increase in sarcomeres. Pathological ventricular hypertrophy due to hypertension differs from physiological hypertrophy [18]. However, in the initial stage of HHD, these changes are also compensatory and often present as concentric thickening. Although the ejection fraction remains in the normal range at this stage, a reduction in overall cardiac contractile reserve and global longitudinal strain has occurred [26]. As the afterload pressure continues, the deterioration of cardiac function persists and eventually leads to heart failure [17]. A number of factors, such as neurohormones, metabolism, immunity, and gut microbiota, play important roles in this process. Furthermore, fibrosis emerges as a pivotal consequence of cardiac injury in hypertension. Cardiac fibrosis significantly fuels the progression of HHD and contributes to adverse outcomes [5]. Thus, we also provide an overview of the mechanisms underpinning cardiac dysfunction in HHD (Fig. 1). In addition, we briefly describe the role of the left atrium and right heart in the development of HHD.

### 2.1 Neurohormones

Hypertension disrupts the neuroendocrine system. Neuroendocrine factors have long been considered the main contributors to the development of HHD [25]. Among them, the renin-angiotensin-aldosterone system (RAAS) and catecholamines play a crucial role in the progression of HHD.

The activation of the RAAS is a crucial component of the pathological changes caused by hypertension. Angiotensin II (Ang II) is an important driver of a series of changes in the heart during HHD [18]. Ang II, which binds to angiotensin II type I receptor (AT1R) on the surface of cardiac myocytes, activates the Gα_q_ protein and phospholipase C (PLC), leading to the production of diacylglycerol and inositol 1,4,5-trisphosphate (${\mathrm{IP}}_{3}$). ${\mathrm{IP}}_{3}$ increases intracellular calcium levels, increasing myocardial contractility through the calcium-calmodulin kinase II pathway [18, 27]. Initially, Ang II stimulation enables the heart to cope with higher afterloads. However, chronic stimulation of Ang II through sustained Gαq activation leads to myocardial hypertrophy [28]. Moreover, overexpression of Gαq can induce apoptosis in cardiac myocytes, which may be associated with increased endonuclease activity due to elevated intracellular calcium levels [29]. Inhibitors of Gα_q_ overexpression block the development of pressure overload-induced cardiac hypertrophy [30]. Another study also demonstrated that negative regulation of G protein-coupled receptor-mediated signaling pathways attenuates Ang II-induced cardiomyocyte hypertrophy and cardiac remodeling due to pressure overload [31, 32]. Ang II can also generate reactive oxygen species (ROS) through coupling with Gα_q_, which may further promote HHD [33, 34]. Apart from its direct effects on cardiac myocytes, Ang II can stimulate cardiac fibroblasts to release various cytokines, such as transforming growth factor-beta 1 (TGF-β1) and interleukin-6, promoting local production of Ang II and AT1R expression [35, 36]. Furthermore, the activation of the RAAS also promotes cardiac fibrosis progression. However, current research does not show beneficial effects of RAAS inhibitor treatment in heart failure with preserved ejection fraction (HFpEF) patients [37]. This suggests that RAAS activation may not be the main mechanism underlying the progression to HFpEF in HHD patients [38].

Increased blood pressure activates the sympathetic nervous system, leading to the release of catecholamines. Catecholamines bind to β-adrenergic receptors in cardiac cells [39]. The heart primarily expresses two types of β-adrenergic receptors (βAR), β1AR and β2AR, with β1AR being the most abundant on the surface of myocardial cells and β2AR accounting for 20% of myocardial adrenergic receptors in normal physiological conditions [40]. Current research suggests that excessive activation of β1AR leads to myocardial cell apoptosis and other adverse effects, while β2AR mediates cardiac protection [41, 42]. β1AR activation stimulates adenylate cyclase via Gα_s_, leading to increased cyclic adenosine monophosphate (cAMP) levels, subsequent activation of protein kinase A, and ultimately, upregulation of contractile proteins and calcium ion levels in myocardial cells [40]. However, cAMP also activates exchange proteins activated by cAMP (EPAC) [43]. EPAC1 contributes to the pathological growth of myocardial cells and may promote the transition from myocardial hypertrophy to heart failure, while EPAC2 may be involved in the occurrence of arrhythmias [18].

However, prolonged exposure to elevated blood pressure results in desensitization of adrenergic receptors due to sustained catecholamine stimulation [40]. β-adrenergic receptor desensitization leads to reduced downstream signaling and impaired cardiac functional reserve. This change serves as an important mechanism underlying the development of heart failure. β-adrenergic receptor desensitization is primarily mediated by GPCR kinases (GRKs) [44]. GRKs belong to the serine/threonine kinase family and consist of seven members, with GRK2 and GRK5 being the main regulators of β-adrenergic receptor desensitization in the heart. Inhibition of GRK2 with paroxetine improves hypertension-induced cardiac hypertrophy, dysfunction, and fibrosis in mice [45]. Additionally, in hypertensive patients with depression, those treated with paroxetine exhibited less cardiac remodeling than patients receiving other antidepressant medications [45]. Furthermore, β-arrestins are also involved in β-adrenergic receptor desensitization [46].

Natriuretic peptides (NPs) constitute a class of peptide hormones with blood pressure-regulating functions categorized into three types: atrial natriuretic peptide (ANP), B-type natriuretic peptide (BNP), and C-type natriuretic peptide (CNP) [47]. ANP and BNP are produced and secreted by the heart, while CNP is synthesized by vascular cells [48]. It has been demonstrated that NPs are closely associated with the development of LVH and HF resulting from hypertension [23]. Upon binding of ANP and BNP to natriuretic peptide receptor (NPR), the activation of guanylate cyclase ensues, leading to increased levels of cyclic guanosine monophosphate (cGMP) [47]. This activation initiates downstream processes, including the activation of protein kinase G (PKG) and cGMP-gated ion channels, ultimately reducing intracellular calcium ion levels [47]. This cascade also suppresses signals linked to cardiac hypertrophy. This series of events is thought to be a critical mechanism through which natriuretic peptides exert their anti-cardiac hypertrophy effects [49]. Furthermore, NPs have been found to mitigate the progression of cardiac fibrosis. Knockout mice lacking NPR exhibit a notable increase in myocardial fibrosis, which does not correlate with blood pressure levels [50]. Additionally, NPs can inhibit the proliferation of cardiac fibroblasts induced by Ang II. Current research suggests that the antifibrotic effects of NPs are also mediated through the cGMP-PKG pathway. ANP is susceptible to degradation by neprilysin. Recent studies indicate that inhibiting neprilysin can impede the activation and proliferation of cardiac fibroblasts, which is attributed to the restoration of PKG signaling within fibroblasts [51]. Furthermore, ANP has been shown to stimulate mitochondrial autophagy, enhance mitochondrial function, and lower intracellular oxidative stress levels [52]. Patients with hypertension exhibit elevated levels of NPs [47]. However, prolonged exposure to high levels of NPs can lead to desensitization of NPR [23]. This desensitization weakens the cardioprotective effects of NPs, thereby promoting the occurrence and development of HHD [53].

### 2.2 Metabolic Remodeling

The heart’s unique function demands a substantial amount of energy. Physiologically, 95% of the heart’s adenosine triphosphate (ATP) is produced through oxidative phosphorylation in the mitochondria, while the remaining ATP is primarily derived from glycolysis [54]. Most (>60%) ATP is generated using fatty acids as metabolic substrates, with the rest coming from glucose, lactate, ketone bodies, and other sources [55, 56]. Cardiac contraction is powered by approximately 60% to 70% of the generated ATP [54]. Due to limited ATP reserves in cardiomyocytes, which can be depleted within seconds, any disruption in the energy production process significantly impacts cardiac function [54]. To cope with this characteristic, the myocardium is highly adaptable in its choice and shift of metabolic substrates, despite fatty acids being the primary substrates [57]. Studies indicate that cardiomyocytes rapidly shift to glucose metabolism when the workload increases [58].

Traditionally, it was believed that myocardial substrate preference would shift toward glucose during ventricular hypertrophy and heart failure [59]. However, recent research suggests that cardiac metabolism maintains a great deal of flexibility even in severe heart failure, allowing it to adjust substrate use to accommodate changes in arterial supply and workload [60]. Consequently, myocardial fatty acid uptake may remain unaffected [18]. However, fatty acid oxidation (FAO) is reduced in pathological ventricular myocardial hypertrophy [57]. The imbalance between fatty acid uptake and utilization leads to the accumulation of lipids such as triglycerides, ceramides, and diacylglycerols, which are synthesized from excess fatty acids within cardiomyocytes. While triglycerides are considered harmless, the lipotoxicity of ceramides and diacylglycerols can result in mitochondrial dysfunction and apoptosis [57, 61, 62]. Furthermore, an excess of intracellular fatty acids can promote the development of myocardial insulin resistance [63, 64].

Acetyl coenzyme A carboxylase is the rate-limiting enzyme for fatty acid synthesis, and animal studies have shown that knocking down the acetyl coenzyme A carboxylase 2 gene in cardiac tissues increases fatty acid oxidation, reduces glucose oxidation, and prevents ventricular hypertrophy caused by increased load [65, 66]. Similarly, overexpression of medium-chain acyl-coenzyme A dehydrogenase has been shown to reduce left ventricular hypertrophy caused by increased load [67]. Additionally, increasing dietary fatty acid intake in the context of heart failure reduces glucose oxidation and improves cardiac function [68].

During HHD, early increased glucose utilization can compensate for the energy deficit caused by decreased fatty acid metabolism efficiency. However, as the pathology progresses, the overall metabolic efficiency decreases [18]. Recent studies indicate that myocardial glucose uptake capacity varies at different stages of progression. The glucose uptake rate rises in hearts with mild diastolic and systolic dysfunction but declines significantly in severe cardiac dysfunction [69]. Cardiomyocytes facilitate glucose entry through glucose transporter proteins (GLUT1 and GLUT4), with GLUT4 being the main isoform in adults and GLUT1 in fetuses [70]. Glucose transport via the GLUT1 pathway increases during the development of cardiac hypertrophy [71]. Overexpression of GLUT1 has been found to prevent heart failure due to increased load and attenuate mitochondrial dysfunction [72]. However, it can aggravate cardiac hypertrophy. Conversely, knocking down GLUT4 leads to more severe cardiac hypertrophy and contractile dysfunction [73]. Therefore, increased glucose levels in cardiomyocytes may attenuate cardiac hypertrophy development. Similar to fatty acids, despite increased glucose uptake and glycolysis, the oxidation rate remains unchanged [18]. This results in the accumulation of metabolic intermediates, including glucose-6-phosphate. The buildup of intermediates, in turn, may promote cell growth and protein synthesis, ultimately contributing to the progression of ventricular hypertrophy. This occurs through activating the mammalian target of rapamycin protein complex 1, enhancing the pentose phosphate pathway, and stimulating the hexosamine biosynthesis pathway [74, 75, 76].

Under physiological conditions, ketone bodies provide approximately 10% of the energy source for the myocardium [77]. In contrast, ketone body metabolism is elevated in patients with heart failure cachexia [57]. It has been shown that specific knockdown of succinyl coenzyme A:3 ketoacyl coenzyme A transferase in mouse cardiomyocytes promotes cardiac hypertrophy [78]. Thus, increased ketone body metabolism may be a metabolic adaptation to HHD.

Branched-chain amino acids provide less than 5% of the energy source for the myocardium under physiologic conditions [77]. In contrast, the levels of branched-chain amino acids are similarly increased in patients with HF. Excessive levels of branched-chain amino acids have been shown to inhibit α-ketoglutarate and pyruvate dehydrogenase activity and promote contractile dysfunction [79, 80]. The mechanism by which they are elevated during pathological remodeling is unclear. However, branched-chain amino acids and their metabolites have been shown to activate mammalian rapamycin target proteins, which may lead to alterations in cardiomyocyte anabolism, proliferation, autophagy, and other processes [77].

### 2.3 Immunity and Inflammation

Inflammation and immunity also play important roles in HHD [18, 81, 82]. Previous clinical trials targeting inflammation in LVH and HF drugs have yielded disappointing results [83]. However, the results of the CANTOS trial demonstrated that interleukin (IL)-1β monoclonal antibody can reduce heart failure-related hospitalization and mortality in patients with previous myocardial infarction, demonstrating the potential of inflammation in HF treatment [84]. While the role of inflammation and immunity in HHD and heart failure has long been recognized, the understanding of this process has been limited by the knowledge of the immune environment in the heart and the available research methods. Recent advancements in fate mapping and single-cell analysis techniques have provided valuable opportunities for advancing cardiac immunology [85]. These tools have revealed the heterogeneity of the cardiac immune system, offering new insights into the roles of immunity and inflammation in HHD.

Macrophages, the most abundant immune cells in the heart, constitute 5–10% of the total cardiac cells [85, 86]. The heterogeneity of cardiac macrophages is being unveiled, with human heart macrophages categorized into at least three types based on transcriptomic and functional differences: self-renewing cardiac resident macrophages (${\mathrm{TIMD4}}^{+}$${\mathrm{LYVE1}}^{+}$${\mathrm{FOLR2}}^{+}$${\mathrm{CCR2}}^{-}$ macrophages, ${\mathrm{TIMD4}}^{-}$${\mathrm{LYVE1}}^{-}$${\mathrm{FOLR2}}^{-}$${\mathrm{CCR2}}^{-}$ macrophages) and ${\mathrm{TIMD4}}^{-}$${\mathrm{LYVE1}}^{-}$${\mathrm{FOLR2}}^{-}$${\mathrm{CCR2}}^{+}$ macrophages derived from circulating monocytes [87, 88]. Current research supports the notion that ${\mathrm{CCR2}}^{-}$ cardiac resident macrophages are involved in tissue repair and maintaining cardiac homeostasis [89, 90]. Under normal physiological conditions, these cells are the primary macrophage source in the adult mammalian heart. Conversely, ${\mathrm{CCR2}}^{+}$ macrophages derived from circulating monocytes increase in number in the postinjury heart and contribute to the inflammatory response during cardiac injury [89, 90].

Macrophages have been identified as key contributors to the development and progression of HHD [91]. Earlier animal experiments utilized the nonselective depletion of macrophages through clodronate liposome injection to investigate their role in HHD. One study demonstrated a deterioration in left ventricular ejection function in hypertensive rats following macrophage removal [92]. Conversely, another study found that macrophage depletion attenuated hypertension-induced left ventricular hypertrophy while improving cardiac fibrosis [93]. These studies suggest a potential heterogeneous role of macrophages in HHD. To further elucidate the distinctions among different macrophage sources, Liao *et al*. [94] employed a ${\mathrm{CCR2}}^{+}$ antagonist-based approach to specifically block circulating-derived monocyte infiltration in mouse hearts. The results revealed an initial increase in macrophage numbers in the heart after transverse aortic constriction (TAC), returning to baseline levels at 2 weeks, and a mild increase again in the late phase (4 weeks). These changes in macrophage numbers corresponded to alterations in cardiac function, with the heart exhibiting compensatory hypertrophy early after TAC, followed by progressive cardiac dysfunction and heart failure at 2–4 weeks. The increase in macrophages resulted from the proliferation of cardiac resident macrophages, which facilitated the initial adaptive changes in response to pressure overload, potentially by promoting vascular generation [94]. A recent study of a genetic fate-mapping approach and single-cell transcriptome analysis demonstrated the cardioprotective role of cardiac resident macrophages in HHD [95]. This study also identified insulin-like growth factor-1 (IGF-1) as a potentially significant pathway for resident macrophage-mediated cardioprotection. The subsequent increase in late cardiac macrophages originated from the infiltration of circulating monocytes. However, circulating-derived ${\mathrm{CCR2}}^{+}$ macrophages mediate inflammatory injury and contribute to the deterioration of cardiac function, while reducing their infiltration helps maintain cardiac function. These findings are supported by additional studies, highlighting the potential of targeting ${\mathrm{CCR2}}^{+}$ macrophages as an important intervention strategy in HHD [96].

The activation of macrophages during HHD involves various mechanisms. Recent studies have shown that Ang II can directly promote macrophage activation through a pathway independent of AT1R [97]. This process is facilitated by the pattern recognition receptor dectin-1 on the macrophage surface, and the knockdown of dectin-1 significantly reduces Ang II-mediated cardiac injury. Although direct studies in HHD are lacking, mechanical stress can directly activate macrophages. A study utilizing a mouse model of dilated cardiomyopathy demonstrated that tissue-resident macrophages can sense mechanical stretch via transient receptor potential vanilloid 4 (TRPV4), leading to increased macrophage production of IGF-1, which plays a critical role in cardiac vascular neogenesis [98]. Furthermore, other studies have indicated that mechanosensory ion channels on the macrophage surface, such as PIEZO1, can respond to changes in mechanical stress and initiate downstream proinflammatory pathways [99].

Lymphocytes likewise play an important role in the pathologic process of HHD. Studies have shown that ${\mathrm{CD4}}^{+}$ T cells promote the development of cardiac fibrosis due to pressure load [100]. In a mouse model of pressure overload, the development of cardiac fibrosis coincided with the infiltration of ${\mathrm{CD4}}^{+}$ T cells into the myocardium [101, 102]. The interaction between cardiac fibroblasts and T cells plays an important role in this process. A recent study demonstrated that cardiac fibroblasts induced by interferon-gamma (IFN-γ) can express major histocompatibility complex II (MHCII) and present antigens to ${\mathrm{CD4}}^{+}$ T cells [103]. At the same time, T cells can promote the transformation of fibroblasts to myofibroblasts, thereby facilitating the progression of fibrosis [101]. Meanwhile, the use of T cells expressing chimeric antigen receptor targeting fibroblast activation proteins significantly reduced cardiac fibrosis in the Ang II-induced hypertensive mouse model [104]. Other immune cells, such as dendritic cells, are also involved in the development of HHD [105].

### 2.4 Gut Microbiota

The gut microbiota is crucial in maintaining host health, impacting various physiological functions, including blood pressure regulation [106]. Bacterial metabolites are instrumental in mediating this effect [107]. Moreover, the gut microbiota has emerged as a significant factor influencing hypertension-induced target-organ damage. A recent study demonstrated that germ-free mice exhibit more severe cardiac and renal injury following Ang II-induced hypertension than colonized mice [108]. This highlights the protective role of gut microbes in mitigating hypertension-induced organ damage. Dietary habits significantly influence physiological factors in the host, with the gut microbiota serving as a vital intermediary. Different dietary patterns impact gut microbial metabolites, which are crucial for their physiological functions [109]. For instance, the fermentation of dietary fiber by gut microbes produces short-chain fatty acids, known to lower blood pressure and reduce cardiac hypertrophy and fibrosis [110]. The gut microbiota also influences other host metabolites associated with hypertension and immune system modulation [106].

### 2.5 Fibrosis

Elevated blood pressure can trigger the development of cardiac fibrosis, characterized by excessive collagen buildup in the spaces between cells and blood vessels [17]. In HHD, the distribution of fibrosis can vary at different stages of progression. In the early stages, fibrosis may be localized to the subendocardium or around blood vessels. As HHD advances, fibrosis can extend outward toward the subepicardium, leading to a more diffuse pattern [16, 111]. Fibrosis reduces the flexibility of the ventricles and serves as the underlying mechanism for hypertension-related HFpEF. The presence of higher levels of fibrosis may explain why pathological LVH is resistant to treatment, unlike physiological hypertrophy [23]. Recent studies have shed light on several factors contributing to cardiac fibrosis in hypertension, including the activation of the RAAS [112, 113], inflammation and immunity [114, 115], and mechanical strain [116].

The production and maintenance of the cardiac collagen matrix primarily involve cardiac fibroblasts, which constitute 10–25% of all cardiac cells [86, 117]. These fibroblasts originate from both the endocardium and the epicardium [118]. When activated, fibroblasts can transform into myofibroblasts, which have an increased capacity to produce collagen and are key contributors to tissue fibrosis following cardiac injury [119]. The current studies support the significant role of proinflammatory cytokines and changes in the extracellular matrix composition in converting fibroblasts into myofibroblasts [116, 120, 121]. It should be noted that the role of fibroblasts from different origins in the development of cardiac fibrosis may not be consistent. Recent investigations indicate that endocardial-origin fibroblasts exhibit a preference for responding to cardiac injury caused by pressure overload compared to epicardial-origin fibroblasts [122]. The Wnt signaling pathway promotes the activation of endocardial-origin fibroblasts. Targeted removal of endocardial-origin fibroblasts attenuated pressure overload-induced cardiac fibrosis and improved cardiac function in one study. However, an earlier study suggested that fibroblasts from different origins play similar roles in cardiac hypertrophy resulting from TAC [123]. The reason for this discrepancy may be advances in genetic tracing techniques.

Myofibroblasts express α-smooth muscle actin, which grants them contractile capabilities [124]. Therefore, during the early stages of the disease, myofibroblast production helps maintain cardiac function to some extent. However, these cells exhibit an enhanced ability to synthesize and secrete collagen, and the collagen produced is fortified through cross-linking, making them more resistant to degradation by matrix metalloproteinases [125]. Consequently, collagen production increases while degradation decreases, leading to collagen matrix accumulation and fibrosis formation. Furthermore, once formed, myofibroblasts can secrete TGF-β, triggering the activation of additional myofibroblasts and further fueling the progression of fibrosis [119].

### 2.6 Left Atrial and Right Heart Involvement in HHD

The heart is an integrated system where various components, apart from the left ventricle (LV), play significant roles in the progression of HHD.

Enlargement of the left atrium (LA) is a recognized hallmark of HHD, and LA dysfunction is pivotal in facilitating the transition to HF while serving as a key marker for cardiovascular conditions, such as atrial fibrillation [126, 127, 128, 129]. The mechanisms behind LA involvement in HHD are multifaceted. Hypertension itself can induce atrial myocardial dysfunction and fibrosis. Indications of LA dilation and fibrosis markers are observed in spontaneously hypertensive rats at seven months of age [130]. Similar to the LV, aberrant activation of the RAAS and the adrenergic system in hypertension is instrumental in LA remodeling and fibrosis [131, 132]. The electrophysiologic function of the left atrium is similarly affected, with hypertension-induced changes in atrial myocytes potentially contributing to the development of atrial fibrillation due to alterations in calcium homeostasis [130, 133]. Meanwhile, unfavorable LV characteristics, including increased LV mass and impaired diastolic function, are associated with diminished LA function in HHD patients, potentially exacerbated by higher afterload [134]. In a mouse model with partial stenosis of the ascending aorta, the left atrium exhibited fibrosis, increased susceptibility to atrial fibrillation, and conduction abnormalities, among other changes [135]. Importantly, the neurohumoral functions related to the LA can also have a significant impact on HHD, as evidenced by a clinical trial demonstrating that left atrial appendage closure significantly reduced RAAS activity and lowered systemic blood pressure [136].

Systemic hypertension also impacts the right ventricle (RV), resulting in concentric RV remodeling and impaired RV diastolic function [137, 138]. Numerous clinical studies have linked adverse RV remodeling to an unfavorable prognosis [139, 140, 141]. Right heart involvement may be attributed to pulmonary hypertension secondary to left heart conditions [142]. Similar to the LV, the right heart undergoes a transition from compensation to decompensation as pulmonary artery pressure rises [143]. Right ventricle-pulmonary artery decoupling occurs when right ventricular contractility no longer matches the increased afterload. The precise mechanism by which left heart alterations lead to pulmonary hypertension remains unclear, with possible contributors including endothelial injury and neuroendocrine hormone metabolic disorders [142, 144].

## 3. Classification of HHD and Assessment

HHD encompasses a spectrum of diseases ranging from asymptomatic functional impairment to heart failure, even SCD [16, 145]. Traditionally, HHD is classified into four stages based on clinical symptoms and echocardiographic findings [146]. In stage one, patients are asymptomatic with echocardiographic evidence of left ventricular diastolic dysfunction but no LVH. Stage two involves asymptomatic or mildly symptomatic patients with LVH observed on echocardiography. In stage three, patients present with heart failure with preserved ejection fraction. Stage four is characterized by heart failure with reduced ejection fraction (HFrEF) and left ventricular dilation.

However, clinical observations and animal study evidence suggest that this classification may be arbitrary [3]. Disease progression does not necessarily follow a linear sequence. For example, although left ventricular hypertrophy is considered a significant manifestation of HHD, it is not a mandatory feature. Cardiac hypertrophy is not always the obligatory compensatory response to increased mechanical load [147]. Complications and patient-specific characteristics, such as age, body mass index (BMI), and complications, can also influence the specific manifestations of HHD in the heart [148, 149].

A more rational approach to identifying HHD involves a comprehensive assessment of left ventricular cardiac morphological changes, diastolic function, mechanics, and interstitial fibrosis [150]. Evaluating these indicators relies on advances in assessment techniques.

Detection of morphological changes to assess LV remodeling in the heart is the traditional diagnostic modality for HHD [146]. As the primary assessment tool, echocardiography allows simultaneous monitoring of left ventricular shape, systolic and diastolic function, and mechanical indices [151]. Nevertheless, the main limitation of echocardiography lies in its dependence on skilled and experienced technicians for image quality. Moreover, the diagnostic thresholds for HHD through imaging remain a contentious area [152]. For example, the left ventricular mass index is calculated by adjusting left ventricular mass to the body surface area, but some studies also support using ${\mathrm{height}}^{2.7}$ for correction [151, 153]. The current American echocardiography guidelines recommend thresholds of >115 g/$m^{2}$ for males and >95 g/$m^{2}$ for females to diagnose left ventricular hypertrophy [154]. However, this diagnostic threshold may differ among people of different races [155, 156]. Hence, further research is needed to establish reference standards for diagnosing left ventricular hypertrophy via cardiac imaging that are suitable for diverse ethnic groups.

Cardiac magnetic resonance (CMR) currently serves as the gold standard for evaluating cardiac structure and function in clinical practice [10]. CMR can also provide information about tissue characteristics, enabling clinicians to assess the level of cardiac fibrosis, a crucial factor influencing HHD progression [150]. However, the cost of CMR and practical constraints, such as patients with metallic implants, hinder its widespread use. Computed tomography (CT) also holds value in assessing cardiac structure, but functional evaluation remains challenging with CT [157].

One of the most classical indicators for assessing cardiac systolic function is the left ventricular ejection fraction (LVEF). However, this indicator may not decrease in patients with HHD until they progress to decompensated heart failure [3]. Global longitudinal strain (GLS) and other cardiac mechanics parameters may offer greater sensitivity [158]. While there is currently no gold standard for *in vivo* myocardial strain measurement, speckle-tracking echocardiography has been validated and widely utilized compared to other methods [159]. GLS is impaired in various forms of hypertension, and it indicates changes in cardiac systolic function before alterations in LVEF [158, 160, 161, 162, 163]. Nevertheless, standardization issues exist with myocardial strain indices, influenced by factors such as measurement devices, operator skills, and timing [159]. Therefore, current guidelines do not establish a normal reference range for myocardial strain but emphasize the heterogeneity of measurement results [154]. Another crucial consideration is that, despite the relative insensitivity of myocardial strain indices to changes in afterload compared to LVEF, alterations in afterload can still significantly affect measurement results [3]. This suggests that changes in the measurement indices may originate from variations in blood pressure. The pressure-strain loop (PSL) is an innovative analytical approach that combines speckle-tracking strain imaging with estimated left ventricular pressure, reducing the impact of afterload on measurement results and providing a more accurate assessment of cardiac function [164]. PSL quantifies myocardial work, including four components: the Global Work Index (GWI), the Global Wasted Work (GWW), the Global Constructive Work (GCW), and the Global Work Efficiency (GWE). These indices reflect the total work during left ventricular systole, work not directly contributing to left ventricular ejection, work essentially contributing to ejection, and the ratio of work essentially contributing to ejection-to-total work. Therefore, in addition to assessing systolic function, a comprehensive analysis of these indices offers insights into cardiac mechanical efficiency.

Changes in cardiac mechanical efficiency are crucial alterations associated with the progression of HHD. The core of mechanical efficiency lies in the ratio between systolic work and total energy expenditure, and these changes have been linked to adverse cardiovascular events [165, 166, 167]. Although invasive cardiac catheterization provides the most precise estimation of cardiac work, it remains challenging for widespread clinical application [168]. Currently, there are various methods for estimating cardiac mechanical efficiency, such as the calculation of the GWE via PSL analysis and the ratio of estimated stroke work (represented as the product of systolic blood pressure and stroke volume) to cardiac oxygen consumption (represented as the product of systolic blood pressure and heart rate) [164, 166]. In pressure-strain loop-based analyses, research indicates that hypertensive patients exhibit increased GWI, GCW, and GWW. However, the most significant increase is observed in GWW, leading to a decrease in GWE, suggesting a reduced cardiac mechanical efficiency in HHD [169, 170]. Moreover, several clinical trials have indicated that antihypertensive therapy can improve mechanical efficiency [171, 172].

Since diastolic dysfunction tends to manifest earlier and more commonly in HHD than systolic dysfunction, the assessment of diastolic function holds particular importance in evaluating HHD. Currently, two-dimensional echocardiography and tissue Doppler measurements of E/A and E/e’ ratios serve as the primary methods for diastolic function assessment and have found widespread clinical application [3, 173]. However, recent research indicates that after antihypertensive treatment, the improvement in GLS is greater than that of E/e’, suggesting that GLS may be a more suitable monitoring parameter for antihypertensive therapy in hypertensive patients [174].

Other imaging techniques also contribute to HHD assessment. Recently, positron emission tomography (PET) has shown promise in predicting the risk of hypertension patients developing heart failure. Physiological indicators derived from PET results, which reflect myocardial perfusion adequacy, can effectively identify subclinical HHD [175].

Biopsy and pathological examination are valuable tools for accurately assessing cardiac abnormalities [176]. In patients who have experienced sudden cardiac death due to HHD, pathological examination reveals myocardial cell hypertrophy and cardiac fibrosis [16]. However, it is important to acknowledge the limitations of using pathological examination for the widespread evaluation of HHD due to the restrictive nature of clinical cardiac biopsies. Another significant constraint is the heterogeneity of HHD lesions throughout different regions of the heart [16]. Limited biopsy samples from specific areas may make it challenging to accurately assess the overall extent of HHD pathology [177].

## 4. Potential Treatments for HHD

Currently, the treatment of HHD involves blood pressure control and cardiac remodeling reversal [178]. Lifestyle management is the basis of blood pressure control. Drug therapy is the main strategy for the treatment of HHD. The current first-line antihypertensive drugs mainly include angiotensin-converting enzyme inhibitors/angiotensin II receptor blockers (ACEIs/ARBs), thiazide diuretics, calcium channel blockers, and beta blockers [179]. Due to the advantage of ACEIs/ARBs in reversing ventricular remodeling by combating RAAS, it is recommended to be used to treat HHD over other first-line antihypertensive drugs [180]. Here, we summarized a randomized controlled study and meta-analysis on the drug treatment of left ventricular hypertrophy (LVH) or cardiac remodeling from 2013–2023 (Table 1, Ref. [22, 181, 182, 183, 184, 185, 186, 187, 188, 189, 190, 191, 192, 193, 194, 195, 196, 197, 198, 199, 200, 201, 202, 203, 204, 205, 206, 207, 208, 209, 210, 211, 212, 213]). However, even with medical treatment, patients with HHD still face the risk of progressing to heart failure (HFpEF or HFrEF) and SCD, and the use of antihypertensive drugs is ineffective in reducing the risk of sudden death in hypertensive patients (relative risk (RR): 0.96, 95% confidence interval (95% CI): 0.81–1.15) [16, 214, 215]. Heart failure is the final stage of HHD, necessitating the selection of appropriate drug treatments based on the specific type of heart failure. Furthermore, interventional devices are also effective treatments for heart failure.

**Table 1.** **Summary of randomized controlled study and meta-analysis on the drug treatment of left ventricular hypertrophy (LVH) or cardiac remodeling**.

Study	Country	Study population	Comparison	Length of follow-up	Main finding
Meta-analysis					
Jian-Shu Chen *et al*., 2020 [181]	China	5402 from 49 studies	/	1992–2020	The use of ARB, in antihypertensive therapy could achieve better efficacy then ACEI, beta-blockers and CCB reversing LVH in hypertensive.
Yao Wang *et al*., 2023 [182]	China	984 from 11 studies	/	2019–2022	SGLT-2i have the beneficial effects on reversal of left ventricular remodeling (LVM: SMD –0.23, 95% CI –0.44 to –0.02; LVMI: SMD –0.25, 95% CI –0.38 to –0.12).
Yiwen Wang *et al*., 2019 [183]	China	10,175 from 20 studies	/	2010–2019	ARNI can improve functional capacity and CRR in patients with HFrEF(LVMI: MD –3.25 g/$m^{2}$, 95% CI –3.78, –2.72).
Quênia Janaína Tomaz de Castro *et al*., 2020 [184]	Brazil	1738 from 5 studies	/	By April 2020	The antihypertensive therapy combined with physical exercise practice can reduce LVM (95% CI –21.63 to –1.81) and HR.
Ye Liu *et al*., 2022 [185]	The United States	209 from 5 studies	/	By June 2020	ARB treatment is not associated with reduced LVM nor remarkable LVEF change among patients with hypertrophic cardiomyopathy.
George C Roush *et al*., 2018 [186]	The United States	2299 patients from 27 articles	/	1992–2009	CHIP diuretics surpass HCTZ for reducing LVM and CHIP diuretics reduce LVM 2‐fold more than HCTZ among hypertensive patients.
Dimitrios Patoulias *et al*., 2020 [187]	Greece	212 patients	/	By July 2020	SGLT-2i treatment in patients with T2D has a favorable effect on LVM.
Li-Ya Yang *et al*., 2013 [188]	China	207 from 6 studies	/	By November 2010	ARBs are associated with a greater reduction in LVH in patients on dialysis. however, the combination of ARBs with ACEIs did not show additional benefit to LVH in patients on hemodialysis.
RenJie Lu *et al*., 2016 [189]	China	4935 CKD patients from 12 studies	/	By December 2015	MRA benefits CKD patients in terms of LVMI, MRA treatment versus non-MRA treatment resulted in a significant change of 0.93 SMD in LVM.
FuWei Xing *et al*., 2017 [190]	China	2566 patients with HT and LVH from 41studies	/	By December 2016	FS-β-B showed greater efficacy when compared with diuretic (MD, 13.04; 95% CI, 3.38, 22.59) or CCB (MD, 10.90; 95% CI, 1.98, 19.49). FS-β-B have the beneficial effects on HT and LVH patients.
Yue Yang *et al*., 2016 [191]	China	357 patients from 8 clinical trials	/	By December 2015	The comparison between ACEI/ARB and controls (other antihypertensive drugs or placebo) showed that the former type of drug causes a greater reduction in LVMI with hemodialysis patients.
Elisa Giannetta *et al*., 2014 [192]	Italy	1622 patients from 24 studies	/	2012–2013	PDE5i have an anti-remodeling effect by reducing cardiac mass (–12.21 g/$m^{2}$, 95% CI: –18.85 to –5.57) in patients with LVH.
Randomized Controlled Trial					
Alexander J M Brown *et al*., 2020 [22]	United Kingdom	66 people with T2D, LVH, and controlled blood pressure	dapagliflozin 10 mg once daily *vs*. placebo	12 months	Dapagliflozin treatment significantly reduced LVM in people with T2D and LVH (mean change –2.92 g; 95% CI: –5.45 to –0.38, *p* = 0.025).
Amil M Shah *et al*., 2022 [193]	The United States	457 PARADISE-MI participants	sacubitril/valsartan twice daily *vs*. ramipril 5 mg twice daily	8 months	Treatment with sacubitril/valsartan compared with ramipril after AMI did not result in changes in LVEF or LAV at 8 months.
Balwant Lal *et al*., 2018 [194]	Pakistan	76 patients with LVH	allopurinol *vs*. febuxostat	6 months	Allopurinol was found to be more effective than febuxostatin reducing the LVM and LVH independent of blood pressure.
Valentina Mercurio *et al*., 2020 [195]	Italy	158 patients with MS and LVH	Nutraceutical combination (berberine 500 mg, red yeast rice 200 mg and policosanol 10 mg) once daily *vs*. placebo	24 weeks	Treatment with AP is associated with a significant reduction in LVM in subjects with MS and LVH.
Alvin Chandra *et al*., 2022 [196]	The United States	1025 patients	vitamin D3 (2000 IU/d) and n-3 fatty acids (1 g/d) *vs*. placebo	2 years	Among adults aged ≥50 years, neither vitamin D3 nor n-3 fatty acids supplementation had significant effects on cardiac structure and function after 2 years.
Phillip D Levy *et al*., 2023 [197]	The United States	113 patients with hypertension (systolic blood pressure [BP] >160 mm Hg), increased LVMI, and vitamin D deficiency (<20 ng/mL)	50,000 IU vitamin D3 *vs*. placebo	1 year	The study not find an association between vitamin D supplementation and differential regression of LVMI or reduction in systolic BP.
Mohapradeep Mohan *et al*., 2019 [198]	United Kingdom	68 patients without diabetes who have CAD with IR and/or pre-diabetes	metformin XL (2000 mg daily dose) *vs*. placebo	1 year	Metformin treatment significantly reduced LVMI (95% CI: –2.63 to –0.12, *p* = 0.033) and LVM (*p* = 0.032).
Luigi Gnudi *et al*., 2023 [199]	United Kingdom	45 patients with T2D and CKD	0.5 mcg calcitriol once daily *vs*. placebo	48 weeks	The study did not provide evidence that treatment with calcitriol as compared to placebo might improve LVMI in patients with T2D, mild left ventricular hypertrophy and stable CKD.
Christopher R Gingles *et al*., 2019 [200]	United Kingdom	362 patients with essential hypertension and LVH	allopurinol (600 mg/day) *vs*. placebo	12 months	Treatment with high-dose allopurinol in normouricemic controlled hypertensive patients and LVH is detrimental (LVM: –0.37 ± 6.08 *versus* –3.75 ± 3.89 g; *p* = 0.012).
Mads Ersbøll *et al*., 2022 [201]	Denmark	91 patients with high-risk T2D	empagliflozin (25 mg/day) *vs*. placebo	13 weeks	13 weeks empagliflozin treatment in patients with type-2 diabetes at high CV risk significantly reduced LVM, improved LV geometry and improved diastolic function compared to placebo.
Roland E Schmieder *et al*., 2017 [202]	Germany	114 patients with essential hypertension	sacubitril/valsartan *vs*. olmesartan	52 weeks	LVMI decreased to a greater extent in the sacubitril/valsartan group compared to the olmesartan group from baseline to 12 weeks (–6.36 *vs*. –2.32 g/$m^{2}$; *p* = 0.039) and from baseline to 52 weeks (–6.83 *vs*. –3.55 g/$m^{2}$; *p* = 0.029).
Greicy Mara Mengue Feniman-De-Stefano *et al*., 2015 [203]	Brazil	17 hemodialysis patients	25 mg of spironolactone *vs*. placebo	6 months	The group receiving spironolactone had a LVMI reduction from 77 ± 14.6 g/$m^{2.7}$ to 69 ± 10.5 g/$m^{2.7}$, *p * < 0.04, whereas in placebo group there was an increase from 71 ± 14.2 g/$m^{2.7}$ to 74 ± 17.4 g/$m^{2.7}$. Spironolactone treatment in hemodialysis patients was secure and effective in regression of left ventricular hypertrophy.
Benjamin R Szwejkowski *et al*., 2013 [204]	United Kingdom	66 T2D patients	Allopurinol, 600 mg/day *vs*. placebo	9 months	Allopurinol causes regression of LVM in patients with T2D and LVH (LVM –2.65 ± 5.91 g *vs*. placebo group +1.21 ± 5.10 g, *p* = 0.012 and LVMI to body surface area –1.32 ± 2.84 g/$m^{2}$*vs*. placebo group +0.65 ± 3.07 g/$m^{2}$, *p* = 0.017).
Hirohiko Motoki *et al*., 2014 [205]	Japan	32 hypertensive patients	5 mg of amlodipine/day *vs*. 16 mg of azelnidipine/day	12 months	Azelnidipine has beneficial effects on LVM regression, transmitral flow, tissue Doppler, and LV longitudinal strain that are comparable to those of amlodipine on the same parameters.
Han Li *et al*., 2013 [206]	China	64 PD patients with hypertension	nitrate group *vs*. non-nitrate group	24 weeks	It was concluded that organic nitrates favor regression of LVH in hypertensive patients on chronic peritoneal dialysis (LVMI reduction:nitrate group: 14.6 ± 4.9 g/$m^{2}$ *vs*. non-nitrate group: 10.6 ± 6.7 g/$m^{2}$).
Uğur Abbas Bal *et al*., 2015 [207]	Turkey	22 postmenopausal osteoporotic women	raloxifene 60 mg/day *vs*. control group	6 months	Raloxifene therapy does not affect myocardial hypertrophy in postmenopausal women after 6 months of treatment.
C Moroni *et al*., 2017 [208]	Italy	56 sex-, age- and therapy-matched subjects with essential hypertension and LVH	losartan (100 mg/die) on-top treatment *vs*. control group	3 years	Losartan induced both a significant reduction of LVH and an improvement of LV diastolic function with a subsequent expected beneficial shift on the prognosis.
Giuseppe Derosa *et al*., 2015 [209]	Italy	145 patients in hypertensive, T2D with LVH	lercanidipine, 20 mg/day and losartan, 100 mg/day *vs*. barnidipine, 20 mg/day and losartan, 100 mg/day	6 months	Barnidipine + losartan provided a greater improvement of echocardiographic parameters compared to lercanidipine + losartan. (such as LVMI and so on).
Huan Zheng *et al*., 2016 [210]	China	50 borderline and mildly hypertensive patients	exercise group received a 4 months’ exercise program *vs*. control group	4 months	Four-month exercise training in borderline and mildly hypertensive patients not only decreased their blood pressure levels, but also induced an improvement of exercise capability, left ventricular remodeling and heart rate recovery.
Sushma Rekhraj *et al*., 2013 [211]	United Kingdom	66 patients with IHD and LVH	600 mg/day allopurinol *vs*. placebo	9 months	High-dose allopurinol regresses LVH, reduces LV end-systolic volume, LVM (allopurinol –5.2 ± 5.8 g *vs*. placebo –1.3 ± 4.48 g; *p* = 0.007) and LVMI (allopurinol –2.2 ± 2.78 g/$m^{2}$ *vs*. placebo –0.53 ± 2.5 g/$m^{2}$; *p* = 0.023).
Yasuhiko Ito *et al*., 2014 [212]	Japan	158 patients under treatment with ACEI or ARB and undergoing peritoneal dialysis	The 25-mg once-daily dose of spironolactone *vs*. control group	2 years	In this trial, spironolactone prevented cardiac hypertrophy and decreases in left ventricular ejection fraction in patients undergoing peritoneal dialysis, without significant adverse effects.
Takafumi Okura *et al*., 2013 [213]	Japan	53 patients with hypertension	50 mg/day of losartan and 12.5 mg/day of HCTZ *vs*. a standard dose of ARB and CCB	48 weeks	These results suggest that combination therapy of an ARB and diuretic has greater potential to cause regression compared with an ARB and CCB.

ARB, angiotensin receptor blockers; ACEI, angiotensin converting enzyme inhibitors; CCB, calcium channel blockers; SGLT-2i, sodium-glucose cotransporter 2 inhibitors; LVM, left ventricular mass; LVMI, left ventricular mass index; SMD, standardized mean difference; 95% CI, 95% confidence interval; ARNI, angiotensin receptor-neprilysin inhibitors; CRR, cardiac reverse remodeling; HFrEF, heart failure with reduced ejection fraction; MD, mean difference; HR, heart rate; LVEF, left ventricular ejection fraction; T2D, type-2 diabetes; CHIP diuretics, chlorthalidone, indapamide, and potassium-sparing diuretic/hydrochlorothiazide; HCTZ, hydrochlorothiazide; MRA, mineralocorticoid-receptor antagonists; LVH, left ventricular hypertrophy; CKD, chronic kidney disease; HT, hypertension; FS-β-B, fat-soluble and selective β1-receptor blockers; PDE5i, phosphodiesterase type 5 inhibitors; AMI, acute myocardial infarction; LAV, left atrial volume; AP, armolipid plus; MS, metabolic syndrome; BP, blood pressure; CAD, coronary artery disease; IR, insulin resistance; CV, cardiovascular; LV, left ventricular; PD, peritoneal dialysis; IHD, ischemic heart disease.

### 4.1 RAAS Inhibitors for HHD Treatment

ACEIs/ARBs are the most commonly used class of drugs for the treatment of HHD, with the effects of lowering blood pressure, reversing cardiac remodeling, and relieving myocardial fibrosis [216, 217]. ACEIs are more recommended than ARBs for the treatment of hypertension [179, 218]. However, in a recent network meta-analysis of randomized controlled trials comparing the effects of different antihypertensive drugs on reversing LVH, ARBs not only have a better effect on reversing LVH than β-blockers and calcium channel blockers but also have a better effect on reversing LVH than ACEIs. Similar advantages of ARBs have been observed in animal studies [219]. This may be because ARBs reduce oxidative stress and better inhibit collagen synthesis [220]. In addition, ARBs reduce cardiac damage by raising plasma Ang II levels and activating the ACE2-angiotensin (1-7) [Ang (1-7)]-Mas pathway [221].

The ACE2-angiotensin (1-7) [Ang (1-7)]-Mas pathway can antagonize the systematic biological effects of the RAAS and become a possible target for the treatment of myocardial fibrosis. An animal study revealed that pirfenidone inhibited the AT1R/p38 MAPK/RAS axis and activated the ACE2-angiotensin (1-7) [Ang (1-7)]-Mas pathway by activating liver X receptor alpha (LXR-α), thereby improving the imbalance of the ACE/ACE2 ratio in rats with myocardial infarction and inhibiting myocardial remodeling and fibrosis [222]. A phase II clinical trial assessing the treatment of HFpEF patients with pirfenidone has shown effective reductions in myocardial fibrosis, but further trials are needed to verify this [223].

Angiotensin receptor/neprilysin inhibitors (ARNIs, such as sacubitril/valsartan) can antagonize both the natriuretic peptide system and RAAS, and they are recommended to be used to treat heart failure in preference to ACEIs/ARBs [224, 225]. Sacubitril/valsartan also effectively reduces blood pressure in hypertensive patients and has a high safety profile [226, 227]. The PARAMETER study revealed that ARNIs are more effective than ACEIs/ARBs in reducing dynamic central aortic and brachial artery pressures in elderly patients with high systolic blood pressure [228]. This suggested that ARNIs are superior to ACEIs/ARBs in controlling and preventing the progression of HHD. However, whether ARNIs can reverse hypertensive LVH is still being investigated (NCT03553810). Therefore, more research is needed to determine whether patients with HHD can use angiotensin receptor-neprilysin inhibitors (ARNIs) as an early alternative to ACEIs/ARBs. In HFrEF, sacubitril/valsartan outperformed ACEIs/ARBs in reducing the risk of cardiovascular death and improving LVH [229]. In HFpEF, a recent meta-analysis showed no significant difference in all-cause and cardiovascular mortality with RAAS antagonists in HFpEF patients, but ARNIs demonstrated superiority over ARB (odds ratio (OR): 0.80, 95% CI: 0.71–0.91) in reducing HFpEF-related hospitalizations [230]. Therefore, when patients with HHD progress to heart failure, ARNIs may be more effective than ACEIs/ARBs.

Mineralocorticoid receptor antagonists (MRAs) are a class of drugs that act on mineralocorticoid receptors by antagonizing aldosterone, which is used to treat heart failure and relieve cardiac remodeling and myocardial fibrosis [231]. In a randomized controlled study of 140 patients with type-2 diabetes, the eplerenone-treated group had a 3.7 g/$m^{2}$ reduction in indexed left ventricular mass from baseline (95% CI: –6.7 to –0.7; *p* = 0.017) [232]. Compared with the control group, the concentration of plasma N terminal pro B type natriuretic peptide (NT-proBNP) and pro-collagen type I N-terminal propeptide decreased significantly, which suggested that it could reverse cardiac remodeling and prevent heart failure [232]. For HFrEF, MRA is often used in combination with other drugs, which are thought to reduce the risk of death better and reverse cardiac remodeling [233, 234]. For HFpEF, MRA does not reduce the risk of death [235, 236]. Therefore, the use of MRA is more beneficial when HHD progresses to HFrEF compared to when it progresses to HFpEF. The use of steroidal MRA (spironolactone, eplerenone) has been limited due to adverse effects (hyperkalemia, renal insufficiency, etc.) [237]. As a nonsteroidal MRA, finerenone is considered safer and can be used for treating diabetic nephropathy and heart failure [237, 238]. At natriuretic doses, finerenone was more effective than eplerenone in reducing cardiac hypertrophy and BNP and improving left ventricular systolic and diastolic function [239]. Finerenone may be an alternative to traditional MRA in the treatment of HHD.

In addition to drug therapy, RAAS antagonism has become the goal of many hypertension vaccines [240, 241, 242, 243]. In animal models, angiotensin I and II vaccines reduced blood pressure in hypertensive rats [240, 241]. However, in clinical trials, angiotensin I, while increasing angiotensin-1 antibody titers, did not reduce blood pressure in patients [242]. A randomized controlled trial evaluating the angiotensin-2 vaccine AGMG0201 revealed that AGMG0201 improved angiotensin-2 antibody titers and had a good safety profile but did not assess the effect of the vaccine on blood pressure in patients [243]. Another recent clinical trial found that zilebesiran, as a small interfering RNA (siRNA) drug, inhibits angiotensinogen synthesis in liver cells, and a single subcutaneous injection can reduce serum angiotensinogen and blood pressure levels and can be maintained for up to 24 weeks [244]. A new clinical trial of zilebesiran as an add-on treatment for patients with inadequately controlled hypertension is also underway (NCT05103332). Due to the lack of long-term antihypertensive drugs, compliance in patients with hypertension and HHD is often low. This often leads to unsatisfactory curative effects for HHD patients. Hypertension vaccines and siRNA drugs are expected to become effective long-term therapies to reduce blood pressure and compensate for the gap in hypertension treatment.

### 4.2 Beta-Blockers for HHD Treatment

The ESC/ESH (European Society of Cardiology/European Society of Hypertension) guidelines for hypertension include beta-blockers as first-line antihypertensive agents, while the ACC/AHA (American College of Cardiology/American Heart Association) guidelines do not [179, 218]. Beta-blockers should be preferred for heart rate control in patients with myocardial infarction [245]. There is a lack of recommendations for the use of beta-blockers in hypertensive LVH without coronary heart disease, possibly due to the limited effect in reversing LVH [246, 247]. However, beta-blockers are superior to other antihypertensive medications in alleviating myocardial ischemia and preventing tachyarrhythmia in hypertensive LVH [246, 247]. Thus, beta-blockers possibly offer irreplaceable advantages in the treatment of HHD. In hypertensive heart failure, beta-blockers can effectively improve patients’ ejection fraction [248]. Moreover, a meta-analysis revealed that beta-blockers may reduce the risk of cardiovascular death in HFpEF patients, but the evidence is limited [235]. Therefore, trials of beta-blockers in the treatment of each stage of HHD are insufficient.

### 4.3 Inhibitors of Sodium-Glucose Cotransporter 2 for HHD Treatment

Cardiometabolic abnormalities are an important risk factor for hypertension and cardiovascular diseases, and patients with metabolic syndrome have a higher risk of HHD [249]. Improving metabolic abnormalities in HHD patients is a new therapeutic target. SGLT2 may be an important target for the treatment of cardiovascular diseases by improving metabolism. In a randomized controlled clinical trial, 124 hypertension patients with type-2 diabetes were randomly given 25 mg empagliflozin quaque die (QD) or a placebo for 12 weeks. The 24 h mean ambulatory blood pressure decreased significantly in patients treated with empagliflozin [250]. Another meta-analysis also demonstrated that SGLT2 inhibitors reduced ambulatory blood pressure but also revealed that the antihypertensive effect of SGLT2 inhibitors was more pronounced during the day than at night and was independent of the dose used [251]. SGLT2 inhibitors are beneficial in patients with LVH. In a randomized controlled trial, a group with type-2 diabetes and LVH using dapagliflozin had a significant decrease in left ventricular mass compared with a control group (95% CI: –5.13 to –0.51 g; *p* = 0.018), effectively reversing left ventricular remodeling [22]. Another randomized controlled trial of the effects of empagliflozin on left ventricular mass in patients with type-2 diabetes and coronary artery disease reported similar results [252]. In hypertensive heart failure rats, empagliflozin can modify cardiac relaxation and contraction functions and alleviate myocardial fibrosis [253]. In other clinical studies, SGLT2 inhibitors have benefits for both HFrEF and HFpEF, mainly reducing the risk of cardiovascular death and hospitalization for heart failure and improving the quality of life of patients with heart failure [254, 255, 256]. More clinical trials of SGLT2 inhibitors in patients with heart failure are also being conducted (NCT05737186). However, there is still a lack of clinical studies on the therapeutic effects of SGLT-2 inhibitors in patients limited to hypertensive heart failure.

### 4.4 Improving Gut Microbiota for HHD Treatment

Probiotics and prebiotics have beneficial effects on patients with HHD through multiple channels. Treatment with Bifidobacterium breve can reduce blood pressure and damage to related target organs in deoxycorticosterone acetate-salt rats [257]. By feeding Lactobacillus fermentum to male Wistar rats on a high-fat diet, systolic blood pressure (SBP) can be effectively reduced, and cardiometabolic abnormalities associated with hypersympathetic function can be inhibited [258]. The role of probiotics and prebiotics in treating hypertension has been further confirmed in clinical trials. In a meta-analysis of 14 studies involving 846 people with high blood pressure, the use of probiotics reduced SBP by 2.05 mmHg (95% CI: 3.87–0.24; *p* = 0.03) and diastolic blood pressure (DBP) by 1.26 mmHg (95% CI: 2.51–0.004; *p* = 0.047) [259]. The intake of multiple probiotics and probiotic intake for more than 11 weeks may have a greater antihypertensive effect [260]. A meta-analysis that explored the effect of prebiotics on blood pressure came to a similar conclusion that the intake of prebiotic beta-glucan fiber was effective in reducing blood pressure [261]. In addition to blood pressure control, probiotics and prebiotics can improve myocardial lesions. The probiotic Lactobacillus rhamnosus GR-1 reduced left ventricular hypertrophy and improved the ejection fraction in rats after myocardial infarction [262]. A high-fiber diet improves gut microbiota and supplements with acetate to lower blood pressure and improve LVH due to myocardial fibrosis and hypertension [263]. In addition, probiotics and prebiotics are also associated with benefits in lipid regulation and obesity treatment [264, 265]. Trials are underway to explore more different combinations of probiotics to treat hypertension [266]. As an important method to improve intestinal flora disorders, fecal microbial transplantation is being explored for more indications [267]. Trials of fecal microbiota transplantation for the treatment of hypertension are also in progress (NCT04406129, NCT05608447) [268].

### 4.5 Lifestyle Modification for HHD Treatment

Lifestyle management is essential for the treatment of hypertension and HHD. The lifestyle benefits for HHD patients mainly include reasonable diet control, moderate physical exercise, prevention of obesity, and moderate alcohol consumption or abstinence [179, 218].

Reasonable diet control is beneficial to patients with hypertension and heart disease. First, sodium intake is considered to have a causal relationship with hypertension, and reasonable sodium intake can effectively prevent and control hypertension [269, 270]. In addition, an appropriate increase in potassium-containing foods can also help control blood pressure [271]. The Dietary Approaches to Stop Hypertension (DASH) diet is considered a dietary strategy for hypertension independent of sodium intake [272]. A meta-analysis of 30 randomized controlled studies (5545 participants) found that the DASH diet reduced SBP by 3.2 mmHg (95% CI: –4.2 to –2.3; *p* < 0.001) and DBP by 2.5 mmHg (95% CI: –3.5 to –1.5; *p* < 0.001), regardless of whether participants had high blood pressure [273]. In addition, the Mediterranean diet can also reduce blood pressure and the risk of heart failure, but there is still insufficient research evidence [274, 275]. Long-term adherence to other dietary strategies, such as the Paleolithic diet (PD) or a Nordic Nutrition Recommendation (NNR) diet, is thought to improve left ventricular remodeling and may also benefit HHD [276].

Proper aerobic exercise can also be beneficial for people with high blood pressure. In a randomized controlled study on the effects of aerobic exercise on patients with refractory hypertension, 24-hour ambulatory blood pressure and office systolic blood pressure in patients with refractory hypertension who engaged in aerobic exercise decreased significantly compared with the control group [277]. Another meta-analysis highlighted that aerobic exercise reduced ambulatory blood pressure only in hypertensive patients taking antihypertensive medications [278]. Exercise is also beneficial for left ventricular hypertrophy in HHD patients. An animal experiment found that the metalloproteinase 2 of rats with losartan combined with physical exercise decreased significantly compared with that of sedentary spontaneously hypertensive rats using losartan. The reduction in cardiomyocyte diameter was observed only in rats with high doses of losartan (10 mg/kg) combined with physical exercise [279]. Another meta-analysis showed that physical exercise combined with antihypertensive medication significantly reduced left ventricular mass (95% CI: –21.63 to –1.81 g), and although there was a downward trend in left ventricular mass index and an upward trend in ejection fraction, the difference was not statistically significant [184].

### 4.6 MicroRNAs for HHD Treatment

MicroRNAs (miRNAs) are a class of endogenous noncoding small RNAs that mainly play a regulatory role in mRNA translation and degradation [280]. Many miRNAs participate in hypertension and HHD development [281, 282, 283]. The method of using microRNA to treat diseases is introducing miRNA-mimic or anti-miRNA oligonucleotide inhibitors [283]. A study has shown that recombinant adeno-associated virus delivery of endogenous and exogenous miRNA-21 into spontaneous hypertensive rats can reduce blood pressure and reverse cardiac hypertrophy. This is related to miRNA-21’s ability to promote mitochondrial translation to produce mitochondrial DNA (mtDNA)-encoded cytochrome b [284]. Another study effectively improved cardiac remodeling and cardiac function in rats with hypertension-induced heart failure by subcutaneous delivery of microRNA-208A inhibitors [285]. However, there is currently a lack of clinical trials on miRNAs for hypertension and HHD, so the efficacy and safety of miRNA therapy remain uncertain.

### 4.7 Interventional Devices for HHD Treatment

When HHD advances to severe heart failure, medication therapy alone is insufficient. Cardiac resynchronization therapy (CRT) is an effective surgical treatment to improve the prognosis and quality of life of patients with heart failure [286, 287, 288]. In recent years, cardiac contractility modulation (CCM) has emerged as a new surgical modality for the treatment of heart failure and has become a significant option for individuals who are ineligible for or do not respond well to CRT. A prospective study identified the safety and efficacy of CCM for HFpEF [289]. This discovery could offer new hope for HHD patients progressing to HFpEF, as they lack effective therapeutic drugs. In addition, patients with HHD usually have a higher risk of ventricular arrhythmia, especially in the presence of LVH and heart failure [290]. Implantable cardioverter defibrillators (ICDs) can effectively treat ventricular arrhythmias and significantly reduce the risk of death in heart failure patients [291, 292]. Therefore, for hypertensive heart failure patients who meet the indications, ICD therapy is necessary.

## 5. Perspectives

The incidence of HHD has been continuously increasing in recent years, with a total of 18.6 million people suffering from HHD worldwide in 2019 and more than one million people dying from HHD each year [4]. The worldwide prevalence of hypertension is still high, and a large proportion of patients with diagnosed hypertension have unsatisfactory blood pressure control, indicating that they are potentially at high risk for HHD [293, 294, 295]. First, due to the symptoms of early hypertension being covert, many hypertension patients have no obvious symptoms, even though some of them have structural changes in the heart [296]. Obvious symptoms typically do not occur until symptoms associated with severe cardiac impairment are present. Second, the heart is one of the most common target organs for hypertension damage [179, 297]. In addition, hypertension is a necessary condition for HHD, and the risk factors for hypertension also promote the development of HHD. However, there are increasing factors that promote and exacerbate hypertension (such as obesity and alcoholism) with changes in nutrition and lifestyle [249, 298, 299, 300].

At present, early diagnosis and treatment of hypertension and control of blood pressure within the normal range are important measures to reduce the occurrence and development of HHD, which mainly includes regular use of appropriate antihypertensive drugs and improvement of lifestyle. In addition to drug therapy and lifestyle improvement, surgery may be used for patients with HHD progressing to HF. However, there is a lack of specific drug therapy for the progression of heart damage in the intermediate process from hypertension to HF. Currently, most patients only control damage in the heart by controlling blood pressure, which is far from sufficient for the treatment of HHD.

## 6. Conclusions

In summary, future studies are needed to determine the mechanism of HHD progression and design drugs specifically to improve hypertension-induced heart damage. In addition, in treating patients with hypertension and HHD, the importance of controlling multiple hypertension risk factors in combination with anti-HHD drugs should be emphasized to improve myocardial remodeling in HHD treatment.
